# Multi-Walled Carbon Nanotube-Doped Tungsten Oxide Thin Films for Hydrogen Gas Sensing

**DOI:** 10.3390/s100807705

**Published:** 2010-08-17

**Authors:** Chatchawal Wongchoosuk, Anurat Wisitsoraat, Ditsayut Phokharatkul, Adisorn Tuantranont, Teerakiat Kerdcharoen

**Affiliations:** 1 Department of Physics and Center of Nanoscience and Nanotechnology, Faculty of Science, Mahidol University, Ratchathewee, Bangkok 10400, Thailand; E-Mail: g5037004@student.mahidol.ac.th; 2 Nanoelectronics and MEMS Laboratory, National Electronics and Computer Technology Center, Klong Luang, Pathumthani 12120, Thailand; E-Mails: anurat.wisitsoraat@nectec.or.th (A.W.); ditsayut.phokharatkul@nectec.or.th (D.P.); adisorn.tuantranont@nectec.or.th (A.T.); 3 NANOTEC Center of Excellence at Mahidol University, National Nanotechnology Center, Thailand

**Keywords:** WO_3_, hydrogen sensor, nanochannels, E-beam evaporation, carbon nanotube

## Abstract

In this work we have fabricated hydrogen gas sensors based on undoped and 1 wt% multi-walled carbon nanotube (MWCNT)-doped tungsten oxide (WO_3_) thin films by means of the powder mixing and electron beam (E-beam) evaporation technique. Hydrogen sensing properties of the thin films have been investigated at different operating temperatures and gas concentrations ranging from 100 ppm to 50,000 ppm. The results indicate that the MWCNT-doped WO_3_ thin film exhibits high sensitivity and selectivity to hydrogen. Thus, MWCNT doping based on E-beam co-evaporation was shown to be an effective means of preparing hydrogen gas sensors with enhanced sensing and reduced operating temperatures. Creation of nanochannels and formation of p-n heterojunctions were proposed as the sensing mechanism underlying the enhanced hydrogen sensitivity of this hybridized gas sensor. To our best knowledge, this is the first report on a MWCNT-doped WO_3_ hydrogen sensor prepared by the E-beam method.

## 1. Introduction

Hydrogen (H_2_) is one of the most useful gases, being used in many chemical processes and various industries including aerospace, medical, petrochemical, transportation, and energy [1–3]. In recent years, H_2_ has attracted a great deal of attention as a potential clean energy source for the next generation of automobiles and household appliances due to its perfectly clean combustion without any release of pollutants or greenhouse gases [4]. However, this low molecular weighted gas can easily leak out and may cause fires or explosions when its concentration in air is between 4% and 75% by volume [5]. Moreover, H_2_ is a colorless, odorless and tasteless gas that cannot be detected by human senses. Therefore, it is very essential to develop the effective H_2_ gas sensors for monitoring of H_2_ leaks.

Tungsten Oxide (WO_3_) is one of the most widely studied gas-sensing materials due to its fast, high sensitivity response toward NO_x_ [6–9], H_2_S [10–13], C_2_H_5_OH [13,14] CO [15], NH_3_ [15–19] and O_3_ [20]. In case of H_2_ detection, it is well known that H_2_ molecules are not activated on the smooth WO_3_ surface of single crystals [21]. Addition of some noble metals such as Pt, Pd, or Au [22–26] to WO_3_ usually improves the sensitivity and selectivity to H_2_ gas. These metal doped WO_3_ films can be prepared by several methods, including screen printing [22], sputtering [23,24] and sol-gel process [25,26].

In the present work, multi-walled carbon nanotube (MWCNT)-doped WO_3_ thin films fabricated by an electron beam (E-beam) evaporation process and their application for H_2_ gas sensing are reported for the first time. The E-beam process offers extensive possibilities for controlling film structure and morphology with desired properties such as dense coating, high thermal efficiency, low contamination, high reliability and high productivity. MWCNTs were selected for doping because of their larger effective surface area, with many sites available to adsorb gas molecules, and their hollow geometry that may be helpful to enhance the sensitivity and reduce the operating temperature. Furthermore, MWCNTs were reported to be sensitive to H_2_, with good recovery times [27].

## 2. Experimental

### 2.1. Preparation of Materials

**Figure 1.**
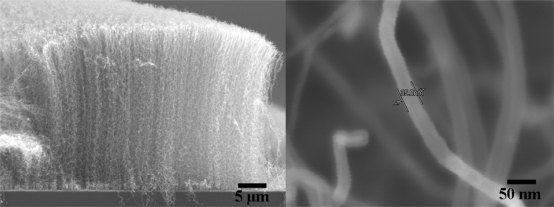
SEM images of the produced MWCNTs grown by the CVD process.

**Figure 2.**
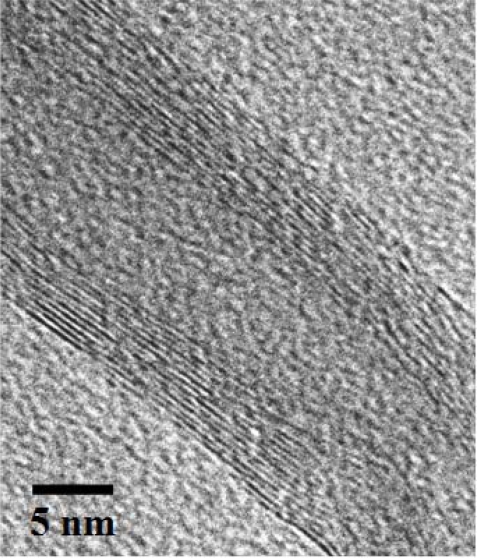
High resolution TEM image of the produced MWCNT grown by the CVD process.

Commercial WO_3_ powder was obtained from Merck and used without further purification. MWCNTs were grown by the thermal chemical vapor deposition (CVD) process. The catalyst layer of aluminium oxide (10 nm) and stainless steel (5 nm) was deposited on the silicon (100) substrates (Semiconductor Wafer Inc.) using reactive sputtering apparatus. The synthesis of MWCNTs was performed under a flow of acetylene/hydrogen at a ratio of 3.6:1 at 700 °C for 3 min. To obtain high-purity MWCNTs, the water-assisted selective etching technique [28] was applied after each CNT’s growth stage. Water vapor (300 ppm) was introduced into the system by bubbling argon gas through liquid water at room temperature for 3 min. The sequence of acetylene/hydrogen and water vapor flows was repeated for five cycles. Based on the scanning electron microscopic (SEM) image, as shown in Figure 1, the diameter and length of the MWCNTs are ∼35 nm and ∼26 μm, respectively. The electrical conductivity of MWCNTs was ∼75 S/cm, as measured by a four-point probe method at room temperature. In addition, high-resolution transmission electron microscopic (HR-TEM) imaging, as shown in Figure 2, confirms that CNTs are multi-walled, with the width and number of walls being ∼4.6 nm and 14, respectively. Thus, the spacing between two graphitic layers is ∼0.33 nm, which is in good agreement with theoretical and experimental values.

### 2.2. Fabrication of MWCNTs-doped WO_3_ Thin Film

MWCNT-doped WO_3_ thin film was fabricated by the E-beam evaporation technique onto Cr/Au interdigitated electrodes on an alumina substrate [29]. The target was prepared by mixing 99 wt% of WO_3_ powder with 1 wt% of MWCNT powder using a grinder in a mortar for 30 min and then pelletizing with a hydraulic compressor. Deposition was performed at a pressure of 5 × 10^−6^ Torr in the evaporation chamber. The substrate was rotated and kept at 130 °C during the deposition in order to obtain a homogeneous thin film. The deposition rate was 2 Å/sec and the final film thickness was 150 nm, as controlled by a quartz crystal monitor. After E-beam evaporation, the film was annealed at 500 °C for 3 h in air to stabilize the crystalline structure. In addition, an undoped WO_3_ thin film was also fabricated using the same conditions for comparison.

### 2.3. Measurement of Gas Sensing

To evaluate the gas sensing properties of the thus prepared thin films, MWCNT-doped WO_3_ and undoped WO_3_ gas sensors were placed inside a stainless steel chamber and the resistance measured using a 8846A Fluke multimeter with 6.5 digit resolution. The gas sensing measurements were made within a dynamic flow system with control of sensor operating temperatures (200–400 °C) under variable gas concentrations (100–50,000 ppm). Hydrogen (H_2_), ethanol (C_2_H_5_OH), methane (CH_4_), acetylene (C_2_H_2_), and ethylene (C_2_H_4_) were used to test the sensing properties and selectivity of the thin films. The sample gas flow time and the clean air reference flow time were fixed at 5 min and 15 min, respectively. It should be noted that these switching interval was selected so that the resistance change is at least 90% of the saturated value. The sensor resistances were sampled and recorded every second using LabVIEW with a USB DAQ device for subsequent analyses.

## 3. Results and Discussion

### 3.1. Characterization of Thin Films

**Figure 3.**
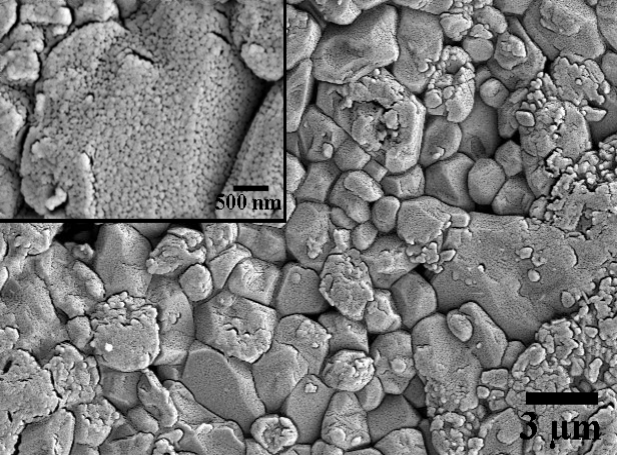
SEM image of MWCNT-doped WO_3_ thin films on alumina substrate.

Surface morphology, particle size and crystalline structure of the films were characterized by SEM and TEM. Figure 3 shows the SEM surface morphology of MWCNT-doped WO_3_ thin film deposited on an alumina substrate. It was seen that the film coated on the rough alumina substrate has approximate grain sizes ranging from 40 to 80 nm.

**Figure 4.**
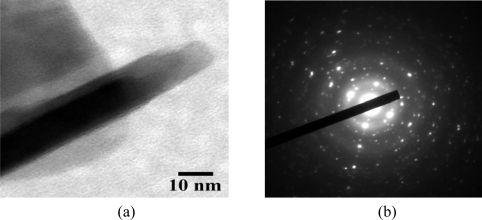
**(a)** High-resolution TEM image and **(b)** corresponding selected area diffraction pattern of MWCNT-doped WO_3_ thin film.

The nanometer grain size together with the roughness of the alumina substrate can enhance the gas sensitivity of thin films [30,31] because more gas adsorption sites are available due to the increased surface area and porosity. With the SEM resolution, CNT structure cannot be observed on the thin film surface. Therefore, TEM characterization was used to confirm CNT inclusion into the WO_3_ film. It should be noted that copper TEM grid samples were loaded inside the evaporation chamber for sample deposition at the same time as coating on the Cr/Au interdigitated electrodes. TEM observation clearly shows CNT inclusion into the nanocrystalline WO_3_, while the electron diffraction pattern exhibits polycrystalline phase in the film, as shown in Figure 4a,b, respectively.

The film morphology obtained in our study is in accordance with observations on nanocrystalline WO_3_ films grown by other methods [32,33]. Doping of CNT does not change the phase or surface morphology of the film, but it may help form nanochannels in WO_3_ films, leading to the enhancement of the sensitivity and reduction of the operating temperature.

### 3.2. Sensing Properties of Thin Films

**Figure 5.**
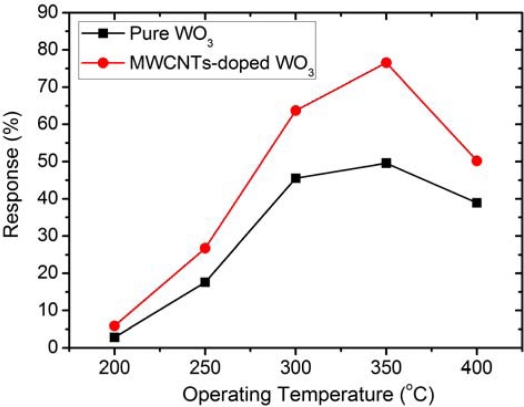
Sensing response to H_2_ (1,000 ppm) at different operating temperatures.

The sensor response (S) of the thin films is defined as the percentage of resistance change:

(1) $$S(\%)=\left(\frac{R_{0}-R}{R_{0}}\right)\times 100$$

where R_0_ and R are the resistance of the thin films in pure air and test gas, respectively. Figure 5 shows the response of the undoped WO_3_ and MWCNT-doped WO_3_ thin films to 1,000 ppm H_2_ at varying operating temperatures. It can be seen that the response of the films increases as the operating temperature increases up to 350 °C, and then decreases. The gas-sensing response increases with temperature in the 200–350 °C range because thermal energy helps the reactions involved overcome their respective activation energy barriers [34,35]. However, if the operating temperature becomes too high (*i.e.*, >350 °C), the adsorbed oxygen species at the sensing sites on the film surface will be diminished and less available to react with H_2_ molecules [36], thereby limiting the film’s response.

At any operating temperature, the sensor response of the MWCNT-doped WO_3_ thin film is higher than that of the undoped WO_3_ thin film. Specifically, at the optimum operating temperature (350 °C), MWCNT-doped WO_3_ thin film yields a 26.9 % higher response than the undoped one. The doped sensor prepared in this work also shows higher response than the WO_3_ films prepared by the sol–gel process [25].

**Figure 6.**
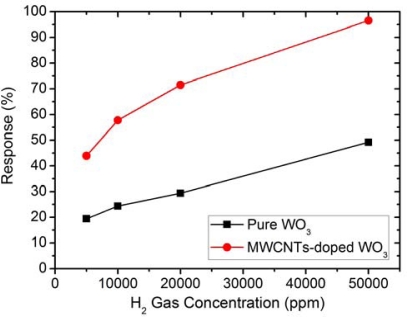
Sensing response of the undoped WO_3_ and MWCNT-doped WO_3_ thin films to high H_2_ concentrations (5,000–50,000 ppm) at the operating temperature of 250 °C.

One major advantage of MWCNT-doped WO_3_ thin film is that the sensor can be operated at a lower operating temperature (250 °C), especially if this sensor is used to measure the H_2_ gas at higher concentrations (5,000–50,000 ppm). As shown in Figure 6, at such a concentration range, there are sufficient numbers of H_2_ molecules available to react with the surface oxygen adsorption sites. It is also well-known that MWCNTs contribute to the reduction of sensor resistance of metal oxides [37] and the activation energy between the WO_3_ surface and H_2_ gas. The details of the sensing mechanisms of MWCNT-doped WO_3_ thin films will be discussed in the next section.

**Figure 7.**
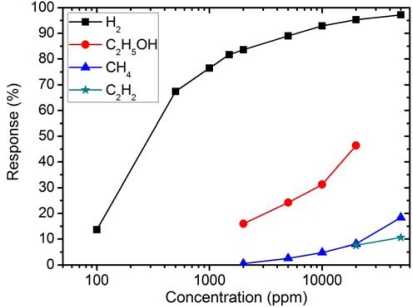
Sensing response of MWCNT-doped WO_3_ thin film at the operating temperature of 350 °C to various concentrations of different gas vapors.

To demonstrate the selectivity of the MWCNT-doped WO_3_ thin film, its sensing response (at the operating temperature of 350 °C) to various gas vapors, namely H_2_, C_2_H_5_OH, CH_4_, and C_2_H_2_, was measured and plotted (Figure 7). It can be seen that MWCNT-doped WO_3_ thin film exhibits a strong response to H_2_, and much weaker responses to C_2_H_5_OH, CH_4_, and C_2_H_2_. In particular, this thin film was found to be insensitive to C_2_H_4_ at the optimum operating temperature of 350 °C. It is therefore concluded that the MWCNT-doped WO_3_ thin film exhibits high selectivity to H_2_.

### 3.3. Sensing Mechanism of MWCNTs-doped WO_3_ Thin Film

It is well known that WO_3_ is an n-type semiconductor while CNT is a p-type semiconductor. MWCNT-doped WO_3_ thin film can be either p-type or n-type semiconductors depending on the quantity of MWCNTs and the operating temperature [38]. In this work, the produced MWCNTs-doped WO_3_ thin film behaves as an n-type semiconductor since the electrical conductivity of the film increases when reducing gases, *i.e.*, H_2_, are absorbed by its surface. Doping of MWCNTs into the WO_3_ matrix can introduce nanochannels and form p-n heterojunctions in the thin film. These nanochannels play an important role for gas diffusion. The gas molecules can easily transport into the gas-sensing layers leading to increasing sensitivity [39,40]. In addition, MWCNT-doped WO_3_ thin film p-n heterojunctions could be formed at the interface between WO_3_ and the MWCNTs [38,41]. When H_2_ gas is exposed to MWCNT-doped WO_3_ thin film, the widths of the depletion layers at the p-n heterojunctions can be modulated. The potential barriers at the interfaces or inside the WO_3_ may be changed. This change of the depletion layer in the p–n heterojunctions of MWCNT-doped WO_3_ thin film may explain the enhanced response of the film at low operating temperatures. Various oxygen species chemisorbed at the thin film surface such as O^2−^, O_2_^−^, and O^−^ are available for catalytic reactions with H_2_, thus depending on the temperature at the metal oxide surface [42]. At the operating temperature range of 200–400 °C, O^−^ is commonly chemisorbed. Consequently, the chemical reaction underlying the H_2_ gas sensing in this study is given by [43]:

(2) $$H_{2}+O_{\mathrm{ads}}^{-}\to H_{2}O+e^{-}$$

The adsorbed O^−^ on the thin film surface reacts with the H_2_ gas yielding H_2_O and releasing electrons which contribute to the current increase through the thin film that causes the electrical conductivity to increase.

## 4. Conclusions

MWCNT-doped WO_3_ thin film was successfully prepared by the E-beam evaporation technique. The 1 wt% MWCNT-doped WO_3_ thin film exhibits n-type semiconductor behavior of the polycrystalline phase. Doping with MWCNTs does not significantly change any phase or surface morphology of the film, but it introduces nanochannels and form p-n heterojunctions in the WO_3_ matrix. The MWCNT-doped WO_3_ thin film exhibits high selectivity and sensitivity to H_2_ over a relatively wide range of concentrations (100–50,000 ppm). Moreover, it can operate at a relatively low temperature. This should be useful for developing high performance H_2_ gas sensors. To our best knowledge, this is the first report on MWCNT-doped WO_3_ hydrogen sensors prepared by the E-beam method.
